# Wireless sequential dual light delivery for programmed PDT in vivo

**DOI:** 10.1038/s41377-024-01437-x

**Published:** 2024-05-15

**Authors:** Jiayi Liu, Bowen Sun, Wenkai Li, Han-Joon Kim, Shu Uin Gan, John S. Ho, Juwita Norasmara Bte Rahmat, Yong Zhang

**Affiliations:** 1 Department of Oncology, The Second Xiangya Hospital, Central South University, Changsha, Hunan 410011 China; 2 Department of Biomedical Engineering, College of Design and Engineering, National University of Singapore, Singapore, 117585 Singapore; 3 Department of Mechanical Engineering, College of Design and Engineering, National University of Singapore, Singapore, 117576 Singapore; 4 Department of Electrical and Computer Engineering, College of Design and Engineering, National University of Singapore, Singapore, 117583 Singapore; 5 Department of Medical IT Convergence Engineering, Kumoh National Institute of Technology, Gumi, 39253 Republic of Korea; 6 Department of Surgery, Yong Loo Lin School of Medicine, National University of Singapore, Singapore, 119228 Singapore; 7 The N.1 Institute for Health, National University of Singapore, Singapore, 117456 Singapore; 8 Institute for Health Innovation and Technology, National University of Singapore, Singapore, 119276 Singapore; 9 Department of Biomedical Engineering, College of Engineering, City University of Hong Kong, Kowloon, Hong Kong SAR, China

**Keywords:** Lasers, LEDs and light sources, Electronics, photonics and device physics

## Abstract

Using photodynamic therapy (PDT) to treat deep-seated cancers is limited due to inefficient delivery of photosensitizers and low tissue penetration of light. Polymeric nanocarriers are widely used for photosensitizer delivery, while the self-quenching of the encapsulated photosensitizers would impair the PDT efficacy. Furthermore, the generated short-lived reactive oxygen spieces (ROS) can hardly diffuse out of nanocarriers, resulting in low PDT efficacy. Therefore, a smart nanocarrier system which can be degraded by light, followed by photosensitizer activation can potentially overcome these limitations and enhance the PDT efficacy. A light-sensitive polymer nanocarrier encapsulating photosensitizer (RB-M) was synthesized. An implantable wireless dual wavelength microLED device which delivers the two light wavelengths sequentially was developed to programmatically control the release and activation of the loaded photosensitizer. Two transmitter coils with matching resonant frequencies allow activation of the connected LEDs to emit different wavelengths independently. Optimal irradiation time, dose, and RB-M concentration were determined using an agent-based digital simulation method. In vitro and in vivo validation experiments in an orthotopic rat liver hepatocellular carcinoma disease model confirmed that the nanocarrier rupture and sequential low dose light irradiation strategy resulted in successful PDT at reduced photosensitizer and irradiation dose, which is a clinically significant event that enhances treatment safety.

## Abstract (web summary)

A light-sensitive polymer nanocarrier encapsulating photosensitizer was synthesized. An implantable wireless device delivers two light wavelengths sequentially was developed to programmatically control the release and activation of the loaded photosensitizer.

## Graphical abstract

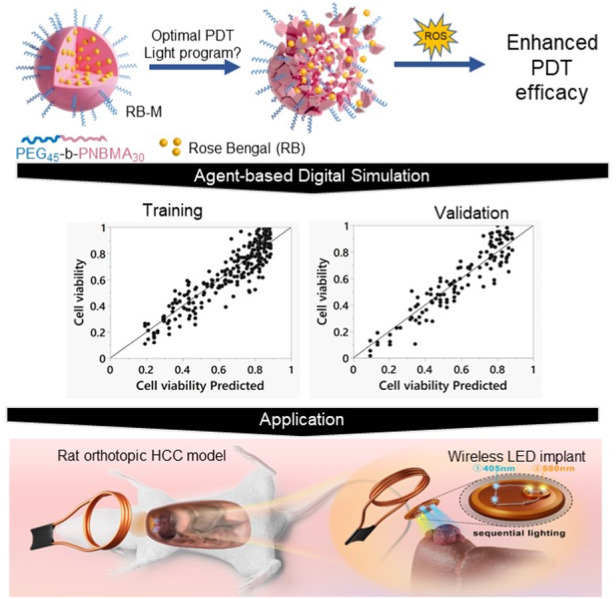

## Introduction

Photodynamic therapy (PDT) is a treatment modality that is highly efficacious when performed in optimum conditions. PDT involves the activation of photosensitizers with a light stimulus, leading to the generation of reactive oxygen species (ROS) which can destroy cellular organelles, resulting in massive damage and death by apoptosis or necrosis, leading to tumor eradication^1,2^. The treatment is also precise since light irradiation can target the tumor site. However, several factors limit PDT efficacy. These include low light penetration ability in deep tissues and low accumulation of photosensitizers in tumor sites^3,4^. To circumvent these challenges, our research group has been working on implantable wireless light delivery systems for repeated PDT of in vivo deep tumors^5–7^ and nanotherapeutics for enhanced photosensitizer delivery^5,8,9^.

Since we first reported using an implantable micro light-emitting diode (LED) device as a light source for in vivo PDT, there has been an increase in the efforts to design similar technologies for cancer treatment^10–12^. Even though dual wavelengths were reported for in vivo use, the device activation process is usually from one energy source performed using a single wireless activation step to power both LED wavelengths. For example, our previous work involved the fabrication of two LED wavelengths (405 nm and 660 nm) on a flexible printed circuit boards (PCB) activated by a radiofrequency signal at 50 MHz^5,7^. The most recent work with implantable light delivery used four microLEDs assembled on a 5-µm thick polyimide substrate transferred onto an injection guide as a needle-like device. However, the four microLEDs are activated concurrently with a wired power source. Additionally, a part of the implant was externalized to allow for wired powering, increasing the risk of infections^10^. Hence, to improve upon previous designs, we fabricated a wirelessly powered dual wavelength LED implant to allow for the controlled and tunable delivery of light of two different wavelengths in deep tissues. In the current upgrade, the two wavelengths used (405 nm for polymer degradation and 580 nm for photosensitizer activation, two microLEDs per wavelength) were fixed on separate PCBs with distinct resonance frequencies to allow the activation of each wavelength independent of each other. Hence, this design incorporates flexibility in the light programs used for multi-step PDT. The two wavelengths can be activated using different input power supplying each wavelength at a different dose.

There has been considerable interest in designing stimuli-responsive nanocarriers that can disintegrate and release the payload^13–17^. We developed a nanocarrier system comprising light-responsive polyethylene glycol-block-poly(4,5-dimethoxy-2-nitrobenzyl methacrylate) (PEG_45_-b-PNBMA_30_) polymeric micelles loaded with RB photosensitizer, designated as RB-M. Nanocarrier disruption was achieved with 405 nm exposure and RB activation at 580 nm for cancer PDT. Combined with the dual PCB implantable microLED device, independent light activation allows for the flexibility of designing light protocols that can result in maximum PDT efficacy. The meticulous control of photosensitizer release from polymeric micelles before light irradiation is important for the following reasons: (1) The ROS generation efficacy of photosensitizers loaded in the nanocarriers would be limited due to self-quenching, (2) the generated ROS may not diffuse out of the polymer nanocarriers or are trapped within the nanoparticle structure, limiting the PDT efficacy, and (3) the gradual release of the photosensitizers from the polymeric micelles regulated by the polymer structure and characteristics will reduce the dose of available photosensitizers for PDT in the tumor tissue. Thus, controlling the release of photosensitizers using stimuli-responsive materials is a rational solution, and light-responsive polymers are the most logical option for PDT applications.

Designing optimal light programs using traditional trial-and-error methods is a time-consuming process. It is difficult to predict the parameters for optimal treatment outcomes based on observations from laboratory experiments alone due to the complexity of biological systems. In conventional clinical PDT, treatment parameters, such as optical power and photosensitizer dosage, are traditionally decided based on empirical evidence. However, such practices led to sub-optimal PDT responses and side effects, and subsequent protocols were adjusted based on currently available clinical data^18–20^. Hence, protocol improvement cycles are slow, leading to the sluggish progress of PDT-based modalities in clinical practice. Computational methods could be adopted to expedite the optimization process as they provide a fast and flexible tool for determining treatment outcomes. Here, we employed an agent-based digital simulation that could model the complex relationships among the factors based on our in vitro experimental results and calculate the priority of each factor for the PDT treatment efficacy, providing data for a clearer understanding of parameters that yield optimum PDT results. Agent-based simulation is a form of machine learning-based inference modeling that has been successfully used to predict brain cancer response to tyrosine kinase inhibitors (TKIs) and to simulate the dynamics between melanoma tumor cells and the immune system in their response to dendritic cell vaccine and 5-fluorouracil co-treatment^21,22^. In our PDT treatment strategy, agents execute various events based on the system they represent: cells, photosensitizers, and light. The digital simulation we performed confirmed our hypothesis. We then validated the simulation results by performing PDT efficacy analysis using the simulation-selected light protocol on 3D liver spheroids and a rat orthotopic liver hepatocellular carcinoma (HCC) model with the synthesized RB-M and the upgraded dual wavelength wireless microLED device.

## Results

### Nanocarrier synthesis and characterization

Light-responsive block copolymer PEG_45_-b-PNBMA_30_ was synthesized using the atom-transfer radical polymerization (ATRP) reaction between PEG-Br and light-responsive monomer 4,5-dimethoxy-2-nitrobenzylmethacrylate (NBMA). The chemical structure and ^1^H nuclear magnetic resonance (NMR) spectrum of PEG_45_-b-PNBMA_30_ are shown in Fig. S1 with the assignments of the corresponding peaks. The degree of polymerization (DP) of NBMA was calculated from the following:

1 $${DP}=\left(\frac{{Ib}}{6}\right)\bigg/\left(\frac{{Ia}}{180}\right)$$

Where Ib is the integral value of the proton peaks of methoxy on the 4, 5-dimethoxy-2-nitrobenzyl group of the PNBMA block (*δ* = 3.39 ppm) and Ia is the integral value of the proton peaks attributed to the PEG block (*δ* = 3.64 ppm). The DP value calculated from the ^1^H NMR spectrum (33) is in good agreement with the theoretical value (30), which confirmed the successful synthesis of PEG_45_-b-PNBMA_30_ block copolymer.

**Fig. 1:**
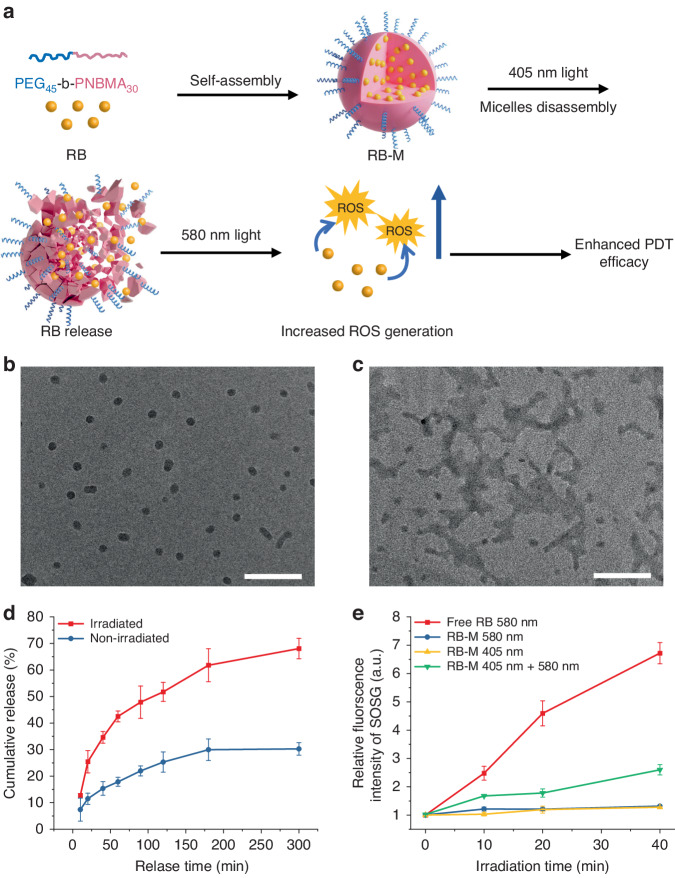
Synthesis and characteristics of RB-loaded light-responsive micelles (RB-M). **a** Schematic illustration of assembly and light-induced disassembly of RB-M. TEM images of RB-M before (**b**) and after (**c**) irradiation from 405 nm light for 40 min (2 mW cm^−2^). Scale bar, 200 nm (**d**) RB release profile from RB-M with and without 405 nm LED irradiation (2 mW cm^−2^) for 60 min. **e** ROS generation by free RB (RB = 10 μM) irradiated with 580 nm LED and RB-M (RB = 10 μM) irradiated with the 405 nm LED alone, 580 nm LED alone, both 580 nm and 405 nm LED together (denoted as 580 nm + 405 nm) respectively as detected by SOSG fluorescence (Ex = 480 nm, Em = 530 nm). Data are presented as mean ± SD (*n* = 3)

It can be hypothesized that PEG_45_-b-PNBMA_30_ self-assembles as nanosized micelles in water due to its amphiphilic nature. Rose Bengal lactone (RB) molecules can be loaded in the core of the micelles via hydrophobic interaction with a loading efficiency (LE) of 14.2% and encapsulation efficiency (EE) of 41.4%. Under 405 nm light irradiation, the *o*-nitrobenzyl groups in the PNBMA block would be cleaved, transforming the latter from hydrophobic to hydrophilic and inducing the degradation of micelles^23,24^. The degradation reduces the interaction between RB and the polymer, thus facilitating the release of RB After RB release, 580 nm light irradiation would activate the RB to generate ROS (Fig. 1a). Photosensitizers loaded in carriers suffer from self-quenching. Furthermore, ROS have a very short life span and diffusion radius^25–27^. Hence, the strategic release and diffusion of photosensitizers within the cellular compartment can increase ROS generation after irradiation and establish the positioning of the photosensitizer at various subcellular organelles for maximum damage, enhancing PDT efficacy. Using light-responsive micelles as a nanocarrier for PDT allows for the controlled release of the photosensitizer. PDT can be achieved by using light of a different wavelength to activate the photosensitizer. The dynamic light scattering (DLS) analysis (Fig. S2) indicated that the hydrodynamic diameter of the RB-M has a narrow size distribution with a Z-average size of 42.8 nm. Transmission electron microscopy (TEM) images of RB-M also showed similar sizes. The structure of RB-M is a spherical micelle with a polymeric core where the RB was loaded (Fig. 1b). After 405 nm irradiation for 60 min, the spherical RB-M degraded. The assemblies changed morphology, which proved the successful cleavage of *o*-nitrobenzyl groups in the PNBMA block (Fig. 1c). In addition, the RB release profile demonstrated that RB-M irradiated with 405 nm LED for 60 min showed higher cumulative RB release than non-irradiated RB-M (Fig. 1d). For example, 68% of RB was released from RB-M irradiated with 405 nm light after a 5 h incubation, while only 30% of RB was released from non-irradiated RB-M after the same incubation duration (Fig. 1d). To verify that the controlled release of RB can enhance ROS generation, the singlet oxygen (^1^O_2_) generated after light activation of free RB and RB-M was assayed using singlet oxygen sensor green (SOSG) reagent. Compared with free RB, which increased the relative fluorescence intensity of SOSG to 6.6× after 40 min irradiation from the 580 nm LED, significantly less ^1^O_2_ was generated from encapsulated RB in RB-M under the same irradiation condition (1.37× SOSG fluorescence) (Fig. 1e). Irradiation with 405 nm LED also induced minimal ^1^O_2_ generation from RB-M because it does not match the absorption spectrum of RB^28^. However, after concurrent irradiation with 405 nm and 580 nm light wavelengths, the fluorescence intensity of SOSG increased to 2.58× after 40 min irradiation (Fig. 1e), albeit lower than free RB. Hence, increased RB release from RB-M after 405 nm light irradiation resulted in enhanced ^1^O_2_ generation with 580 nm irradiation.

#### Fabrication and safety of the wireless device system

**Fig. 2:**
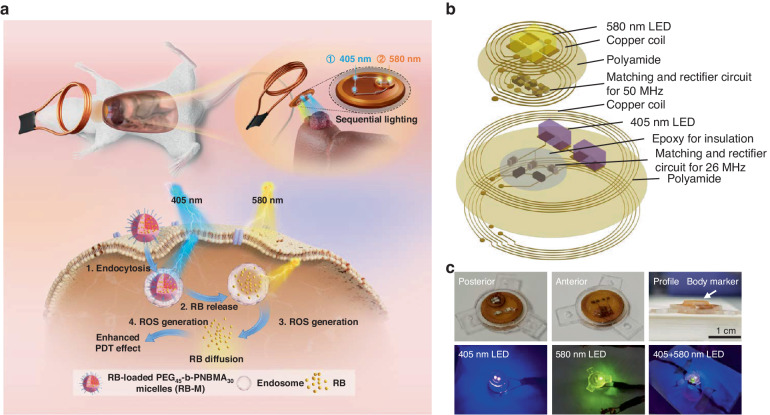
Wirelessly activated microLED implant and sequential PDT treatment. **a** Schematic diagram displaying the sequential light program for enhanced PDT therapy with RB-M nanoparticles. **b** Illustration of 580 nm and 405 nm LED structure. **c** Photos of integrated LED implant and its different lighting modes

A wireless LED device was fabricated as an in vivo light source implanted adjacent to the rat liver tumor (Fig. 2a). The device comprises two PCB that emit violet light at 405 nm and green light at 580 nm (Fig. 2b). The profile display of the electronic components is shown in the supporting information (Fig. S3). Fabrication and characterization of the device were reported in our previous work^5^. The components were double-coated with epoxy and polydimethylsiloxane (PDMS) to protect them from mechanical shock and block the inflow of body fluid. This fixation strategy has been successful in stabilizing the implant components from the mechanical stress of daily movements. Three suture holes were designated to fix the LED device beneath the peritoneum (Fig. 2c). The diameter (without the suture holes component) and height of the device were 10 and 4 mm (589.7 ± 2.4 mg), respectively (Fig. 2c). The wireless transmitter includes a parallel-connected coil array for the concurrent activation of five devices. However, only two arrays were used simultaneously in our in vivo work (Fig. S4). Due to the different designated frequencies of 25 MHz and 50 MHz, activation of the 405 nm and 580 nm LEDs can be independently achieved by the transmitter coil with matching resonant frequency (Fig. 2c), allowing for controlled light irradiation. Tissue temperature change measurements were done on the liver of rat carcasses after wireless powering to find the maximum allowable input power that is safe and will not induce thermal tissue damage. Input power levels at 630 mW and 400 mW were chosen for the 405 nm and 580 nm circuitry, respectively, because the tissue temperature increases were below the threshold that causes thermal tissue damage in the liver^29^. The observed maximum temperature increase was 1.3 ± 0.5 °C for the 405 nm circuitry and 5.2 ± 0.7 °C for the 580 nm LED (Fig. S5).

#### Cell viability and digital simulation

**Fig. 3:**
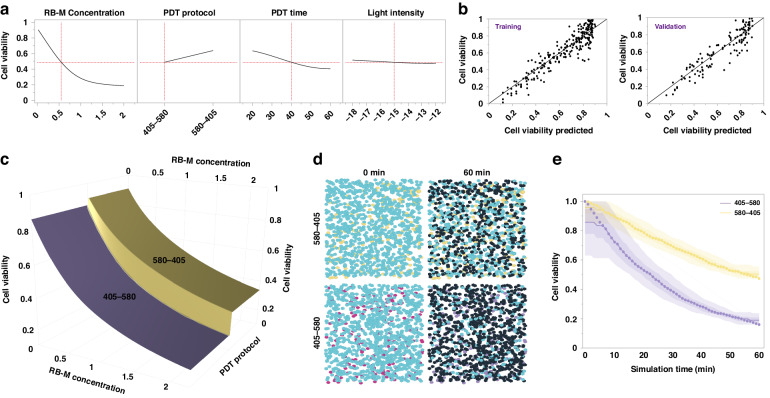
Modeling and simulation of RB-M PDT treatment process. **a** The fitting curve of RB-M concentration, PDT protocol, PDT time, and light intensity using a neural network. RB-M concentration, mg mL^−1^; PDT time, min; Light intensity input power, dBm (−18 = 316.2 mW, −17 = 398.1 mW, −16 = 501.2 mW, −15 = 630.9 mW, −14 = 794.3, −13 = 1000 mW, −12 = 1258.9 mW) (**b**) Observed values of cell viability predicted in training and validation datasets. **c** Relationship between RB-M concentration, selected PDT protocol, and cell viability. **d** The changing dynamics of cell and RB-M agents using different PDT lighting protocols (RB-M concentration: 2 mg mL^−1^, PDT time: 60 min, input power: 400 mW). The cyan cloud indicates active cell agent, the dark gray cloud indicates dead cell agent, the yellow ellipse indicates RB-M PDT treatment with 580–405 protocol, and the purple ellipse indicates RB-M PDT treatment with 405–580 protocol. 580–405 = 580 nm LED irradiation followed by 405 nm LED irradiation. 405–580 = 405 nm LED irradiation followed by 580 nm LED irradiation. **e** Real-time changes in cell viability during simulation

Various parameters are involved in clinical PDT, such as photosensitizer dose, irradiation time, and light intensity. Additionally, irradiation protocols involving two light wavelengths can contribute to PDT augmentation with the RB-M formulation. Hence, to rapidly study the critical factors involved in RB-M PDT, we employed an agent-based digital simulation analysis before further in vitro evaluation. The initial ROS analysis with RB-M solution compared the effects of RB activation alone to concurrent light exposure (Fig. 1e). However, comparison between the two light programs using digital simulation is challenging due to the difference in overall light dose and irradiation time. Furthermore, concurrent exposure of RB-M to both 405 nm and 580 nm light did not induce similar singlet oxygen generation to free RB (Fig. 1e). This observation led to the hypothesis that a sequential protocol incorporating RB release from the micelle before PDT may be more efficacious in generating singlet oxygen and enhancing PDT toxicity effect. Hence, two alternate lighting protocols were used to generate the datasets for the model and to test our hypothesis: 405–580 and 580–405. The protocols involve sequential irradiation of each light wavelength with a timing gap between the light exposure to ensure adequate diffusion of the photosensitizer. The fitting model was generated using a neural network to find the optimum parameters for PDT with RB-M (Fig. 3a). The static snapshot showed the predicted cell viability as a function of different input parameters. As demonstrated by Fig. 3a, RB-M concentration had the most significant effect on cell viability. PDT lighting protocol, time, and light intensity had minimal effects in boosting cytotoxicity. The actual cell viability values on the Y-axis and the model-predicted values on the X-axis for both training and validation datasets are shown in Fig. 3b. The value of R2 and SSE was used to measure the fitting model. R2 was based on the likelihood function and was scaled to have a maximum value of 1, while SSE gives the error sums of squares. The value of (R2, SSE) for training and validation datasets were (0.8545, 1.0947) and (0.8339, 2.3566), separately. The values indicate that the actual cell viability value moves relatively in line with the model-predicted value. As RB-M concentration value increases, the 405–580 protocol outperformed the 580–405 protocol with a faster rate of decline in cell viability (Fig. 3c). Particularly, when the value of RB-M concentration was higher than 1 mg mL^−1^, the difference in cell viability between the two protocols was around 0.2. The difference was further evaluated by an agent-based simulation run (Fig. 3d). The changing dynamics of cell and photosensitizer agents in different PDT protocols were presented. The RB-M concentration value was set to 2 mg mL^−1^, and the LED was wirelessly powered at 400 mW for 60 min. The left part of the Fig. 3d shows the initial, and the right shows the final state of agent crowds for different PDT lighting protocols. Cell and photosensitizer agents were visualized using different shapes, while the different colors represented different states of those agents. After the simulated PDT treatment, the 405–580 protocol displayed lower cell viability at around 0.2, compared with the value of 0.45 generated from the 580–405 protocol simulation (Fig. 3e). Hence, at RB-M concentration greater than 1 mg mL^−1^, the PDT efficacy of the 405–580 irradiation protocol significantly surpassed the 580–405 protocol. The higher in vitro PDT performance of the 405–580 protocol compared with 580–405 can be explained as after the RB-M was internalized, the 405 nm light irradiation would result in the degradation of micelles and the diffusion of loaded RB in the intracellular space. Thus, the following 580 nm light irradiation would enhance PDT results. However, if the cells were irradiated with 580 nm light first, the ROS generation would be suppressed because the RB was trapped in the nanocarriers.

#### RB-M distribution and ROS detection in tumor spheroid

Based on the digital simulation results, in vitro experiments with 3D spheroids used a 2 mg mL^−1^ RB-M concentration to further study RB-M PDT mechanisms. A light irradiation time of 40 min was used as it results in a cell viability decline to below 40% (Fig. 3a). Before advancing with PDT experiments, a time-dependent uptake of RB-M was conducted on HepG2 spheroids to identify the optimal RB-M incubation time for in vitro studies (Fig. S6). Confocal microscopy detection of RB fluorescence showed nanoparticle penetration into the center of spheroids as early as 2 h with more enhanced fluorescence levels at 12 h (Fig. S6A). Fluorescence intensity analysis showed a corresponding increase in RB uptake with incubation time (*p* < 0.01, Fig. S6B) which corroborated with confocal microscopy observations. At 12 h incubation time, the RB-M nanoparticle penetration thoroughly infiltrated the tumor spheroids volume. Henceforth, 3D spheroids were treated with RB-M for 12 h to ensure complete and adequate spheroid infiltration before PDT.

ROS production is a hallmark of a successful PDT. Hence, ROS detection assay after PDT with RB-M was conducted to show that the delivered photosensitizer induced oxidative stress in the presence of light (Fig. S7). For in vitro studies, another irradiation protocol was added to determine if concurrent activation of the 580 nm and 405 nm (580 + 405) light sources could induce PDT toxicity similar to the 405–580 light program. The concurrent light program represents an alternative PDT irradiation protocol in which stimuli-responsive nanocarriers are irradiated simultaneously using both the UV and visible light to activate the polymer nanovesicles and the photosensitizer at the same time. A concurrent light program was added to the investigation henceforth as it is an attractive option in clinical practice due to shorter irradiation timing than sequential lighting.

In the absence of light, the RB-M-only group presented minimal green fluorescence generated from the ROS detection kit, which could result from the auto-activation of the released RB from the nanocarrier. Of the three light programs, only the concurrent 580 + 405 and sequential 405–580 protocols displayed intense green fluorescence covering the whole spheroid area (Fig. S7A). The 580–405 light program-treated spheroid resulted in only minimal green fluorescence. Quantitative assessment of green fluorescence indicated that the light programs induced ROS production in the following order: 405–580 > 580 + 405 > 580–405. Only the 405–580 and 580 + 405 light programs significantly increased green fluorescence intensity compared to the control (Fig. S7B). Furthermore, the means between the 405–580 and 580 + 405 groups were significantly different (*p* < 0.01, Fig. S7B), indicating that the 405–580 sequential lighting protocol could potentially result in enhanced PDT efficacy compared to the concurrent light program.

#### In vitro PDT efficacy on 3D spheroids

Invasion and metastasis are characteristics of HCC diseases that lead to recurrence and poor prognosis after treatment^30,31^. Hence, 3D tumor spheroids derived from HCC cell lines were used for in vitro investigations to examine the effects of RB-M PDT with various light programs on the proliferation, migration, and invasive capacity of the cells in the 3D tumor mass. Calcein AM and ethidium homodimer-1 were used to label live (green fluorescence) and dead cells (red fluorescence) respectively for spheroid viability measurement. Only live cell fluorescence was observed in the control and RB-M-only group (Fig. S8A, C). The red fluorescence from dead cells was visible only in the PDT groups, accompanied by corresponding reduced green signals from live cells (Fig. S8A, C). Out of the three PDT groups, only the concurrent 405 + 580 and the sequential 405–580 light programs resulted in significant toxicity compared to the control (*p* < 0.01, Fig. S8B, D). Furthermore, there is a significant difference between the spheroid viabilities between the two effective PDT groups (*p* < 0.01, Fig. S8B, D).

**Fig. 4:**
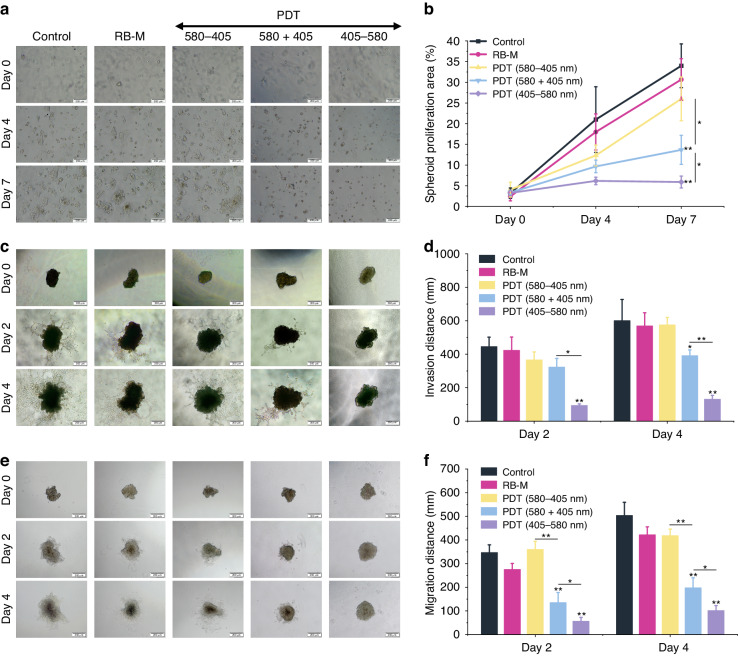
Effects of RB-M PDT treatment on the anchorage-independent proliferation, migration, and invasion of McA-RH7777 3D spheroids. **a** McA-RH7777 spheroid proliferation assay after RB-M PDT using various light programs. Images were obtained at 4× objective Scale bar, 200 µm. **b** Spheroid proliferation area after PDT with various light programs. **c** Bright-field microscopy images of Mca-RH7777 spheroid invasion on Day 0, Day 2, and Day 4 after PDT. Images were obtained at 4× objective Scale bar, 200 µm. **d** Average invasion distance of the cells in the spheroid periphery after PDT. **e** Bright-field microscopy images of Mca-RH7777 spheroid migration at Day 0, Day 2, and Day 4 after PDT. Images were obtained at 4× objective Scale bar, 200um. **f** Average migration distance of the spheroids. Scale bar, 200 µm. Data are presented as mean ± SD (*n* = 3) *p < 0.05, **p < 0.01

The ability of cancer cells to evolve and acquire anchorage-independent proliferation is a crucial step in detachment and metastasis^32^. To study anchorage-independent growth, spheroids after PDT treatment were transferred to a 3D scaffold (Matrigel^®^). Daily microscopic observations showed that spheroids from the control, RB-M-only, and sequential PDT 580–405 groups increased in size steadily in the biological 3D scaffold (Fig. 4a). Similarly, the spheroid invasion distance and migration distance in these groups grew over time (Fig. 4c, e). However, the proliferation, migration, and invasion of spheroids receiving the concurrent 580 + 405 and sequential 405–580 were remarkably inhibited over time (Fig. 4a, c, e). Quantitative analysis of microscopy images verified these trends (*p* < 0.01, Fig. 4b, d, f). Migration assay with 2D Mca-RH7777 cells was performed to validate the migration assay results observed in 3D spheroids. Similarly, only the concurrent 580 + 405 and sequential 405–580 light programs resulted in significantly reduced migration rates (*p* < 0.01, Fig. S9). Interestingly, the spheroids receiving the sequential irradiation protocol 405–580 exhibited the best performance with the least proliferation area, invasion, and migration distance compared with the remaining groups. Further quantitative analysis revealed the statistical differences in proliferation (*p* = 0.021), migration (*p* = 0.039), and invasion (*p* < 0.01) between concurrent PDT 580 + 405 and sequential PDT 405–580 groups (Fig. 4b, d, f). Hence, these two light programs were selected for in vivo evaluation of RB-M PDT efficacy in controlling orthotopic rat HCC tumors.

#### In vivo PDT efficacy

Initially, RB-M was administered at a dose of 1 mg kg^−1^ via the intravenous route, and in vivo tumor mass infiltration was observed for up to 48 h. However, no RB fluorescence was observed in the implanted tumor via an in vivo imaging system (IVIS, data not shown). Hence, RB-M was administered intratumorally via ultrasound guidance injection, resulting in sufficient accumulation of RB signals within the tumor after 2 h, minimal retention at 24 h, and almost complete clearance by 48 h (Fig. S4). Tumor volumes were calculated before RB-M injection, and 0.2 mL of 4 mg mL^−1^ RB-M per 500 mm^3^ tumor volume was administered (1.6 mg cm^−3^ drug dose to tumor volume ratio). After the intratumoral delivery of RB-M, ex vivo IVIS imaging of vital organs was performed to determine the spread of RB within the tumor mass and systemic biodistribution of RB-M (Fig. S10A). At 2 h, the RB-M signals had covered approximately 87.5% of the tumor mass. There was a significant difference in nanoparticle signals between the liver tumor and the remaining organs. In the next 48 h, the RB-M retention rate in liver tumors decreased to below 8.7% over time (*p* < 0.01, Fig. S10A). We did not observe significant fluorescence RB signals in other tissues at 48 h.

**Fig. 5:**
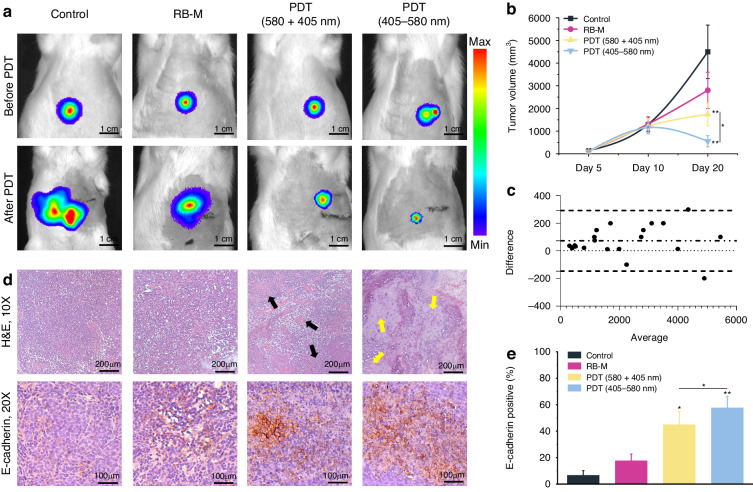
Efficacy and pathological analysis of PDT treatment. **a** IVIS bioluminescence imaging of the molecular beacon (MB) before and after PDT treatment. **b** Quantitative curves of tumor volume by ultrasound detection. **c** Bland-Altman assay analysis of the agreement of tumor volume measured by ultrasound and vernier caliper. **d** H&E and immunohistopathological staining of tumor tissue after PDT treatment. H&E scale bar, 200 µm. E-cadherin scale bar, 100 µm. Black and yellow arrows indicate necrotic regions. **e** Quantitative analysis of E-cadherin positive expression. The data were pooled from two experiments (*n* = 6 for each group). Data are presented as mean ± SD *p < 0.05, **p < 0.01

We developed a 5-day treatment schedule to treat the orthotopic liver tumors. PDT was performed on 3 days with a 1-day break between the doses. Intratumoral injection of RB-M was performed under ultrasound guidance, followed by irradiation with the light programs 2 h later. The tumor length and width were measured using ultrasound imaging for tumor volume calculations. Tumor growth and presence were regularly monitored via IVIS imaging of tumor luminescence signals (Fig. 5a). PDT commenced when tumors reached a minimum volume of 1000 mm^3^ on Day 10 post-tumor implantation (Fig. 5b). The rats were monitored closely for their well-being and signs of early termination criteria (wound abcess, infection, dehiscence, persistent pain, 20% loss of body weight, low body condition score, change in mucous membrane color) due to difficulties of observing tumors on a daily or frequent basis using imaging techniques. Between Day 10 to Day 20, three RB-M PDT sessions were administered to the rats in the sequential and concurent light program groups. Hence, a significant decrease in tumor burden was observed in these groups (Fig. 5b). We conducted computed tomography (CT) scans after the last PDT session to confirm that the positioning of the implanted LED remained secured during treatment (Fig. S4E). After termination, one rat in the control group was observed to have a large tumor (>5000 mm^3^), which was above the recommended tumor size (4000 mm^3^ for subcutaneous tumors) for rats. However, the rat did not display observable signs of early termination criteria and the tumor weight accounted for ~2% total body weight, which posed no significant risk to its well-being. AAALAC guidelines indicate tumor burden of 10% total body weight necessitating immediate intervention or euthanasia^33^.

Based on regular IVIS imaging observations, the control group exhibited exponential tumor growth and presented intrahepatic metastases at the end of the experiment (Fig. 5a). The rats in the RB-M without irradiation group experienced tumor growth similar to the control group, albeit smaller tumors were observed in this group compared to the control by the end of the experiment. The concurrent PDT 580 + 405 and sequential PDT 405–580 groups led to significant tumor regression compared to the control and RB-M-only groups. Means comparison analysis of the tumor growth curve via ultrasound imaging indicated that the concurrent PDT 580 + 405 and the sequential PDT 405–580 groups had significant tumor growth inhibition compared to control and RB-M-only groups (*p* < 0.01). Furthermore, there is a significant difference between the two PDT groups (*p* < 0.01), indicating that sequential 405–580 PDT irradiation exhibited enhanced PDT efficacy compared to the concurrent 580 + 405 light program. After termination, the liver tumors were excised for direct measurements using a vernier caliper as it is considered a standard tool for calculating the tumor volume. Upon termination, a thin film of fibrotic tissue were found surrounding the device (<1 mm). This observation corresponds with our previous experience with this device^5,7^. We also analyzed the tumor volume data consistency between ultrasound and vernier caliper measurements by Bland-Altman assay. The assay confirmed that the bias and 95% limits of agreement of the two methods were 72.85 and 112.3, respectively, which is very close to the baseline, signifying the strong consistency of data between these results (Fig. 5c).

Hematoxylin and eosin (H&E) staining of the tumor tissue sections in the RB-M and control groups harvested at the end of the experiment showed intact tumor cells and intense regions of nucleation, indicating the degree of cellular proliferation in these groups. In the concurrent 405 + 580 light program group, necrotic foci with inflammatory exudation were observed (black arrows, Fig. 5d). Increased necrotic regions (yellow arrows, Fig. 5d) with diffused inflammatory cell infiltrations were observed in the sequential 405–580 group, indicating the higher intensity of damage to the liver tumor cells in this group. The expression of E-cadherin, a transmembrane protein that connects epithelial cells at adherent junctions, was examined by immunohistochemistry analysis. In healthy tissues, high E-cadherin expression exerts a tumor-suppressing role by inhibiting cellular proliferation and facilitating cell-cell interactions, blocking the movement of cells out of the tissue environment^34^. In a meta-analysis study, reduced E-Cadherin expression was identified as a poor prognosis marker for patients with HCC^35^. Hence, the expression levels of this cell adhesion molecule was investigated in this study. Immunohistochemistry staining of tumor tissue slices from the different groups demonstrated increased E-cadherin staining in the PDT groups compared with that of the control and RB-M group (Fig. 5d). Quantitative analysis of the ratio of E-cadherin positive cells in the tissue sections revealed significantly elevated E-cadherin expression in the PDT groups compared to the control group, demonstrating the diminution of the risk of cancer metastasis after PDT treatment. Specifically, the control group exhibited E-cadherin expression at 6.7%, whereas that of RB-M treated group is 17.7%, the PDT groups exhibited the highest E-cadherin expression (45.0% for 580 + 405 nm and 57.7% for 405–580 nm) (Fig. 5e). Additionally, the E-cadherin expression ratios between the sequential 405–580 and concurrent 580 + 405 PDT groups were significantly different (Fig. 5e), indicating the difference in the extent of the PDT effect.

## Discussion

Summarizing our study, we developed a UV-responsive nanocarrier system to engineer the controlled release of photosensitizers for PDT applications combined with an improved implantable microLED device that can independently power two light wavelengths for multi-step therapeutical activations. We also demonstrated a digital simulation model via machine learning to predetermine optimal PDT parameters for cancer treatment. The combination of technologies facilitated the rapid development and optimization of a PDT treatment regimen that successfully controlled and treated implanted tumors in an orthotopic rat liver HCC model, which is one of the few demonstrations of the application of in vivo PDT in deep-seated liver disease^36–38^.

The UV-responsive micelle nanocarrier system we developed enables the flexibility of designing optimal light programs with photosensitizers activatable in the 500–700 nm range. However, delivering UV light in deep tissues is challenging due to its limited penetration depth. Hence, implantable light delivery devices are an attractive option to circumvent this challenge. Here, we developed an improved version of an implantable wireless LED device with dual light wavelengths to facilitate the sequential on-demand light-induced unloading and activation of photosensitizers in deep-seated orthotopic liver tumors. The sequential delivery option is an improvement over most iterations of similar devices because previous versions can only perform concurrent dual wavelength delivery. We have successfully treated in vivo bladder tumors using similar devices in our earlier work^5,7^. Previous research has investigated the application of wireless LED devices for cancer PDT in anatomical locations such as the subcutaneous space, brain, and bladder, allowing the delivery of multiple doses of light from a one-time implant procedure^5–7,12^. Similarly, we demonstrated that a one-time implantation procedure could supply three doses of light for PDT of a deep-seated tumor in a 21-day experiment schedule. Controlled and sequential light delivery was enabled by constructing the LEDs for the two light wavelengths on two separate PCBs with distinct resonant frequencies. This simple yet efficient design upgrade enabled the independent activation of each wavelength using two distinct resonant frequencies (25 MHz for the 405 nm and 50 MHz for the 580 nm). While not the most advanced system, this approach is user-friendly, particularly for individuals without an engineering background. A crucial aspect of this multi-resonant frequency system requires a significant frequency separation of at least twice the operating frequency to ensure independent operation. The short but intense PDT treatment schedule was fashioned after the neoadjuvant chemotherapy schedule for HCC, which depending on the therapy drug, requires daily infusions of 5–28 days due to the aggressive nature of the disease^39,40^. Although the PDT protocol in this study lasted 5 days, we have previously demonstrated that the implant can survive in vivo and remain functional for up to 33 days^5^.

The implantable device we fabricated for in vivo light delivery has multiple advantages. The device is constructed using commercially available inorganic electronics, making them affordable and reliable. However, these electronics tend to be rigid, potentially leading to discomfort and mechanical stress when placed on soft biological tissues. To address this issue, we employed soft biocompatible and transparent PDMS to encapsulate the device. PDMS also acts as a thermal insulator, mitigating unwanted heating effects on the tissue resulting from the electronic components. These characteristics of PDMS are ideal for wireless powering and light delivery^41^. Nevertheless, while PDMS provides benefits, it is not an effective strategy for protecting the device against biofluid exposure and mechanical stress. We applied nontoxic glue to the electronics to address additional protection concerns. The powering system is also straightforward, convenient, and easy to use, which opens the possibility for patients to activate the device themselves in the comfort of their homes. The safety and toxicity of the polymer used for the micelle nanocarrier were established in an earlier publication which used the block copolymers poly(4,5-dimethoxy-2-nitrobenzyl methacrylate)-polyethylene glycol (PNBMA-PEG) to functionalize individual upconversion nanoparticles and to load RB for near-infrared-controlled PDT. The formulation administered at 150 mg kg^−1^ body weight did not induce observable apoptotic or necrotic areas in the histology staining of the brain, heart, liver, and leg muscles of nude mice^24^. For comparison, our RB-M formulation was administered intratumorally at a 2.7 mg kg^−1^ body weight dose in SD rats.

Regarding biosafety, the implantation of the sham device (in the control group) or intratumoral administration of the RB-M alone did not induce apoptosis or necrosis in the excised livers. IVIS imaging monitoring and detection demonstrated clearance of the micelle formulation within 48 h. The presence of the implant in the peritoneal space for 5 days induced the formation of a thin layer (<1 mm) of fibrotic tissue around the encapsulation, which is a normal response to a foreign body. The RB-M administration and implant activation combination was safe and did not induce adverse health effects during treatment. The wireless activation of the 580 nm implanted device resulted in a mild tissue temperature increase in rat carcass (5.2 ± 0.7 °C, Fig. S5A**)**. The temperature increase in live rats were expected to be 1–2°C lower due to the presence of a functioning circulatory system that will dissipate the heat^42^. The mild temperature increase is not sufficient to induce the thermal ablation of the tumor as a monotherapy, which is generally performed at temperatures above 50 °C to induce direct cell death^43,44^. However, the heightened temperature may sensitize the tumor cells to RB-M PDT^45^. Hence, it is plausible that the enhanced therapeutic effect in the PDT groups is attributable to combination of hyperthermia and PDT.

Hence, implantable wireless LEDs combined with RB-M for PDT of HCC diseases are an attractive alternative for recurrent HCC patients with chemo and radio-resistant cancers as there is no mechanistic crossover between the modalities. Clinical PDT protocols can be performed daily to deliver light to the deep-seated tumor after a single implantation procedure, reducing the onerous surgeries required for light delivery with optic fibers. However, more effort is required to miniaturize the device further. It would benefit both patient and physician if the device could be implanted using a needle via a biopsy, as the procedure is simple with faster recovery time. Another future design upgrade option is to digitally program the light activation sequences via an external application interface to enable automation of the PDT sequence. Automation of the irradiation sequence will reduce user associated errors and treatment inconsistencies.

Nanoparticles are internalized by the cells via various endocytic pathways, entrapped in the endosomes, and subsequently degraded by the highly acidic environment in the fused endolysosomal complex^46^. As such, the endosomal escape strategy has been widely investigated to evade the eventual degradation of nanocarriers and their payload^47,48^. In our study, we developed PEG_45_-b-PMBMA_30_ micelles as photodegradable nanocarriers for RB photosensitizer (RB-M). The RB-M nanocarrier system requires a two-step activation procedure using light stimuli: (i) the degradation of the polymer to release the photosensitizer (405 nm) and (ii) the activation of the released RB (580 nm). Although the sequential lighting protocol for PDT using RB-M is more complex than a single continuous irradiation dose used in conventional PDT, it is essential in reducing the desorption of photosensitizers from the micelles. Reduced desorption increases the topical photosensitizer concentration compared to conventional PDT treatment^7,49,50^. In our study, a 1 mg mL^–1^ RB-M solution contains approximately 14.2% loaded RB, equating to 0.014 mg mL^−1^ concentration irradiated at 2 mW cm^−2^ or 4.8 J cm^−2^. In contrast, previous reports using similar formulations used either a higher light dose (21 J cm^−2^) or a higher photosensitizer dose (1 µM) to achieve PDT efficacy^51,52^. Hence, we used a lower RB concentration and irradiation dose than previous reports using polymer vesicles for RB delivery. Thus, accommodating polymer degradation to photosensitizer delivery strategies yields immense benefits in reducing both the photosensitizer and irradiation doses needed for effective PDT.

In the context of treating HCC diseases, nanoparticle encapsulation and controlled release can aid in the retention of photosensitizers in the liver tumor tissue, as free photosensitizers can be pumped out by multi-drug resistant proteins that are highly expressed in HCC diseases^53,54^. Strategic release of RB from encapsulation before activation also renders the photosensitizer effective in lower doses, reducing photosensitizer-related side effects, which is especially important in treating HCC with accompanying cirrhosis^55^. Combined with the degradability of the polymer, which was removed from the system within 48 h, the formulation is safe and does not overburden the diseased liver. However, the administration route limits the application of RB-M formulation as it must be delivered by intratumoral injection. Systemic delivery of drugs and nanoparticles to liver tumors is preferable but is an immense challenge. Several tactics were investigated to improve delivery and retention rates, including active and passive targeting to the liver^56,57^. Hence, RB-M can be further functionalized to endow the formulation with stealth and liver tumor-targeting moieties for convenient drug dispensation. Despite the limitation, RB-M PDT with sequential 405–580 light irradiation program resulted in excellent tumor burden control, with an approximate 2–2.5-fold tumor volume reduction from Day 10 to Day 20 and a 7–9-fold difference in tumor volume size compared to control on Day 20 (Fig. 5b).

Conventional PDT protocols using laser light sources have relied on empirical evidence involving phases of fine-tuning clinical parameters such as photosensitizer dose, light dose, and the start of PDT after photosensitizer administration^18^. However, such protocols are time-consuming and deny patients undergoing PDT the opportunity to be treated with the ideal conditions. In this study, we used an agent-based digital simulation to determine the critical factors involved in PDT with RB-M formulation by multivariate analysis. Above 1 mg mL^−1^ RB-M concentration, the choice of lighting protocol influenced PDT-induced cell toxicity significantly. The digital simulation findings were further validated with in vitro 3D spheroid experiments that yielded the same conclusive results. Two intriguing in vitro results were observed in this study. Firstly, the levels of ROS production and toxicity induced by the sequential 580–405 light program were minimal. With the 580–405 light program, the RB molecules were first photoactivated, followed by the subsequent release of RB to the intracellular space with UV light exposure. ROS were expected to seep from the RB-M particle space, resulting in endosomal rupture, escape, and PDT success. However, the minimal results imply that the ROS generated after 580 nm light exposure could not escape the polymer vesicles and were entrapped, leading to a non-significant PDT effect. Releasing the RB with UV-induced polymer degradation after RB photoactivation does little to improve the results because ROS are short-lived molecules with limited diffusion radius^58^. Hence, active ROS was limited to the polymeric space within the endosomes when RB photoactivation occurred, sequestering the free radicals safely from vital cellular components.

Secondly, the sequential 405–580 light program was significantly more efficacious than the concurrent 405 + 580 program. The enhanced efficacy could be attributed to adequate timing, which allowed the RB to be sufficiently released from the nanocarrier before photoactivation and spread further within the intracellular space, causing maximum damage through their short-lived ROS production after light activation. Unlike the 580–405 light program, which first activates the RB with minimal toxic effects, allowing the RB to escape the polymer before photoactivation yields significant differences in PDT efficacy between the two sequential light programs. Therefore, our findings suggest that establishing a digital simulation model to predict treatment outcomes and determine the critical treatment factors can reduce the extensive characterization work and maximize PDT treatment efficiency.

In recent years, 3D cell spheroid culture models have gained traction as a standard research tool. The main advantage of 3D models over traditional monolayer cultures is their ability to mimic the architecture and heterogeneity of solid tumors. Thus, 3D models have been widely considered a more relevant technique for treatment efficacy studies and are widely used in drug discovery research^59–61^. This article demonstrated HCC spheroid model applications in determining anchorage-independent proliferation, migration, and invasion assays. Our experiments showed the rich manifestations of tumor spheroids, including growth characteristics of anchorage-independent tumor spheroid, interactions between tumor spheroids and extracellular matrix, and spontaneous invasion of tumor spheroids into the matrix. Such assays could demonstrate the phenotypical changes of solid tumors and provide the observational basis for further investigations into the mechanisms of treatment efficacy. From our in vitro 3D spheroid experiments, we can conclude that RB-M PDT with the sequential light program 405–580 eradicated HCC tumor cells by controlling the processes involved in anchorage-independent proliferation, invasion, and migration, all of which are common characteristics of the HCC disease. The histology staining results corroborated with the in vitro spheroid study observations, determining the anti-metastatic effects of the PDT regimen.

In conclusion, this work presents the use of multiple technologies to customize an optimized PDT regimen for curing an orthotopic liver HCC disease. The design improvements of the microLED implant enabled the independent activation of two distinct wavelengths of light for multi-step therapeutics. The microLED activation is tunable to suit the intensity needs of each light wavelength. It can also be easily controlled, allowing repeated PDT of deep-seated tumors. The digital simulation platform provides an innovative method for treatment protocol optimization and has the potential to become a standard for predicting outcomes. The approach can impact treatment outcomes and ensures that patients are treated with an optimized protocol at the start of the therapy, increasing their odds of survival. The collective strategy maximizes treatment potential and minimizes patient discomfort, providing an attractive alternative for a disease that has limited effective options in its treatment arsenal.

## Materials and Methods

### Synthesis and characterization of light-responsive PEG_45_-b-PNBMA_X_ block copolymer

Light-responsive monomer NBMA was first synthesized. In an ice bath, 4,5-Dimethoxyl-2-nitrobenzyl alcohol (2.13 g) was first dissolved in tetrahydrofuran (THF, 40 mL). After the solution was cooled down, 2 mL triethylamine was added to the solution with stirring. Then, 1.5 mL of methacryloyl chloride diluted with 10 mL THF was added slowly through an addition funnel for 30 min. After 2 h of reaction in the ice bath, the mixture was kept at room temperature overnight. After solvent removal by a rotary evaporator, the crude product was dissolved in chloroform and purified by washing with 1 M hydrochloric acid, saturated sodium bicarbonate, and Milli Q water sequentially. Finally, the product was dried with anhydrous magnesium sulfate. After filtration, the filtrate was concentrated into a solid via rotatory evaporation and dried in a vacuum oven at 40 °C overnight. The solid product was then recrystallized in 5 mL methanol and dried in a vacuum oven at 40 °C overnight.

The PEG_45_-b-PNBMA_30_ block copolymer was then synthesized via ATRP reaction. Cu(I)Br (14 mg), poly(ethylene glycol) methyl ether 2-bromoisobutyrate (Mw = 2000) (PEG-Br) (120 mg, 0.06 mmol), NBMA monomer (500 mg, 1.8 mmol), *N,N,N’,N”,N”*-pentamethyldiethylenetriamine (20 µL) and dimethyl sulfoxide (DMSO) (2 mL) were dissolved in a 25 mL Schlenk flask. The mixture was degassed three times using the freeze-pump-thaw procedure and sealed under a vacuum. After 10 min stirring at room temperature, the setup was placed in a preheated oil bath (90 °C) for 24 h. The solution was then precipitated into methanol twice, reprecipitated into ice-cooled diethyl ether twice, then filtered and dried in a vacuum oven overnight. The structure of the synthesized PEG_45_-b-PNBMA_30_ block copolymer was characterized by ^1^H NMR with a 400 MHz Bruker DMX400 spectrometer using CDCl_3_ as solvent.

### Preparation and characterization of RB-loaded PEG_45_-b-PNBMA_30_ micelles

RB-loaded PEG_45_-b-PNBMA_30_ was prepared in the following manner: RB (3.2 mg) was dissolved in 0.5 mL THF. PEG_45_-b-PNBMA_30_ (4 mg) was first dissolved in 1.5 mL THF in a glass vial, and 0.5 mL of the as-prepared THF solution of RB was added to the vial. Then, Milli Q water (8 mL) was added to the solution dropwise at a 4 mL h^−1^. The solution was stirred at room temperature for 2 h before THF was removed by rotatory evaporation at 40 °C. The solution (about 8 mL) was then placed in a 2000 Da dialysis tube and dialyzed against 2 L Milli Q water for 24 h, with frequent water changes during the dialysis. Finally, the product was purified by passing through a 0.45 µm nylon filter and denoted as RB-M. To determine the loading of RB in the micelles, the RB-M micellar solution was freeze-dried and redissolved in DMSO, and the concentration of RB was determined using the Shimadzu UV-2600 UV-vis spectrophotometer with a standard curve of free RB dissolved in DMSO. The LE and EE of RB in the RB-M was calculated as follows:

2 $${LE}=\left({weight}\,{of}\,{RB}\right)/\left({weight}\,{of}\,{RB}\,{loaded}\,{carriers}\right)\times 100 \%$$

3 $${EE}=\left({weight}\,{of}\,{RB}\right)/\left({weight}\,{of}\,{RB}\,{added}\right)\times 100 \%$$

The DLS hydrodynamic diameter and size distribution of the RB-M were measured by a Malvern Zetasizer nanoseries (Malvern Instruments Ltd., UK), and the sizes and morphologies of the RB-M before and after irradiation from 405 nm irradiation 40 min (2 mW cm^−2^) were characterized by TEM using a JEOL-2010-F field emission transmission electron microscope (JEOL Ltd, Japan).

### Establishment of simulation model and multivariate analysis

A network-based model was built to describe the impact of RB-M concentration, PDT protocol, PDT time, and light intensity on cell viability. The cell viability measurement dataset was randomly split into a training (*n* = 252) and validation (*n* = 126) dataset. The network contains a single hidden layer with 20 nodes using hyperbolic tangent as an activation function. The quality of the fitting model is evaluated using the coefficient of determination (R2) and Standard Square Error (SSE) for the training model and the difference between R2 and SSE between the training and validation datasets.

The agent-based modeling approach is developed to simulate the dynamics of the PDT process. This approach allows the simulation of systems with intricate nonlinear relationships that may be hard to describe mathematically. The proposed model consists of agents representing cells and photosensitizers, each with specific attributes and behaviors. A cell agent contains active and dead states; a photosensitizer agent contains three states: inactive, activated by 405–580 protocol, and activated by 580–405 protocol. The simulation processes and updates the states of agents over time. The model takes RB-M concentration, PDT protocol, time, and light intensity as input parameters and produces a response (cell viability). The network-based fitting model is implemented in this simulation to approximate the behavior of components in the simulated system. The fitting model is deployed based on Python and can be queried in the simulation runtime.

### Orthotopic rat liver cancer model and LED implantation

The animal protocols in this study were approved by the Institutional Animal Care and Use Committee, National University of Singapore (Protocol ID: R21-1327). Male SD rats weighing 200–250 g (4 weeks) were purchased from (Invivos, Singapore). Rats were left to rest for 1 week after arrival in the NUS Comparative Medicine facility before start of experiment. About 3 × 10^6^ N1S1-Luc cells were injected into the left liver lobe per rat by percutaneous injection under ultrasound guidance (0.1 mL). The tumor sizes were measured via ultrasound once every 5 days. The formula used for tumor volume calculation is:

4 $${Tumor}\,{Volume}=\left[{Length}\left(L\right)\times {{Width}}^{2}\left({W}^{2}\right)\right]\times 0.5$$

When the tumor volume of N1S1-Luc tumor-bearing rats reached 1000 mm^3^, rats were further divided into four groups (*n* = 6 per group) for further investigation of PDT treatment. Then, LEDs were surgically implanted adjacent to the liver tumor and fixed to the peritoneum (Supporting Information Fig. S4B, C). After surgery and wound closure, the positioning of the LED can be determined by gentle touch to ensure its continued good fixation. Rats were given post-operative care of buprenorphine (0.05 mg kg^−1^, twice daily for 3 days, 0.2 mL per 100 g weight) and enrofloxacin (5 mg kg^−1^, once daily for 5 days, 0.1 mL per 100 g weight) administered subcutaneously. Skin sutures were removed after the wound has completely healed (~10–14 days post-surgery). Rats would be excluded from the data analysis if the LEDs were found to be shifted after surgery during routine care or after post-treatment CT scan. Due to the difficulty of obtaining daily observations of the orthotopic tumor growth via imaging techniques, the rats were observed daily for signs of early termination criteria: hemorrhage, wound abscess, infection, or dehiscence, persistent and severe pain, 20% loss of body weight; low body condition score, change in mucous membrane color (e.g., yellow due to liver failure-induced jaundice), and hypothermia. Termination was performed using CO_2_ asphyxiation followed by bilateral thoracotomy of the euthanized animal.

### In vivo RB-M distribution and PDT efficacy

To investigate RB-M distribution, liver tumor-bearing rats (*n* = 3 per group) with similar tumor volumes were intratumorally injected under ultrasound guidance with RB-M solution at the dosage of 1.6 mg cm^−3^ drug-to-tumor volume ratio (0.2 mL of 4 mg mL^−1^ injected in a 500 mm^3^ tumor volume size). After 2 h, 12 h, and 24 h, the rats were sacrificed. Their tumors and major organs (lung, liver, heart, spleen, kidney) were excised and washed with PBS. An IVIS spectrum imaging system was used to record fluorescence imaging results and calculate average fluorescence intensities.

After LED implantation, the rats were divided into four groups (*n* = 5): (i) sham surgery; (ii) RB-M only; (iii) concurrent PDT 580 + 405; (iv) sequential PDT 405–580. Then, they were intratumorally injected under ultrasound guiding with RB-M solution at the dosage of 1.6 mg cm^−3^ drug to tumor volume ratio in the experimental groups or saline (0.2 mL) in the sham group once every 2 days for a total of three doses. Irradiation with the implanted wireless LEDs was done to initiate PDT treatment after 2 h of RB-M injection. The tumor volume was detected by ultrasound imaging every 5 days. IVIS imaging was concurrently performed by injecting 100 mg kg^−1^ D-luciferin intraperitoneally at 0.2 mL per 100 g weight (Perkin Elmer, MA, USA). A CT scan was randomly performed to detect the location of the LED to ensure that the LED was still located above the liver tumor. On the 10th day of the treatment commencement, the rats were terminated. Before termination, the LED was removed, and the final tumor volume was measured by ultrasound and IVIS. After excision, the tumor volume was verified by the vernier caliper measurements. Bland-Altman assay was used to analyze the data consistency of tumor volume measured by ultrasound and vernier caliper. The cleaned excised tumors were collected and fixed in 10% neutral buffered formalin solution and transferred into 75% ethanol solution after 48 h. For histological staining, the fixed tumor tissue samples were sent to the Advanced Imaging and Histology Core, Immunology Program, Life Sciences Institute, NUS, for tissue processing, embedding, sectioning, and staining. Immunohistochemistry staining with E-cadherin antibody (AB76319-1001, EP913(2)Y, Lot no GR3217511-9), and secondary goat anti-rabbit antibody (AB214880-1001, Lot no GR3440024-1) was performed according to the manufacturer’s protocol (Abcam, Cambridge, UK). The histological sections were examined under an optical microscope (DP73, Japan) and quantified using ImageJ (NIH, USA).

### Statistical analysis

All data except for the simulation model were expressed as mean values ± standard deviation. Statistical calculations were carried out by the Prism software version 9.0. Comparisons between two groups were performed using the student’s *t* test or Mann-Whitney U test. One-way ANOVA was used for the multi-group comparison of means with Bonferroni post-hoc test. Values with *p* < 0.05 are considered significant.

### Supplementary information

Supplementary material
