# Supplementary material for: Wireless sequential dual light delivery for programmed PDT in vivo
Source: Light Sci Appl. 2024 May 15;13:113. doi: 10.1038/s41377-024-01437-x (PMC11094163; doi:10.1038/s41377-024-01437-x)
Supplement: Supplementary file 1 — Supplementary material [file 41377_2024_1437_MOESM1_ESM.docx]

**Supplementary information** for

Wireless sequential dual light delivery for programmed PDT in vivo

**Supplementary information**

**Wireless sequential dual light delivery for programmed PDT in vivo**

Jiayi Liu^1,⊥^, Bowen Sun^2,⊥^, Wenkai Li^3,⊥^, Han-Joon Kim^4,5^, Shu Uin Gan^6^, John S. Ho^4,7^, Juwita Norasmara Bte Rahmat^2,^*, and Yong Zhang^8,^*

^⊥^These authors contributed equally

^1^ Department of Oncology, The Second Xiangya Hospital, Central South University,

Changsha, Hunan 410011, China

^2^ Department of Biomedical Engineering, College of Design and Engineering, National University of Singapore, Singapore 117585, Singapore

^3^ Department of Mechanical Engineering, College of Design and Engineering, National University of Singapore, Singapore 117576, Singapore

^4^ Department of Electrical and Computer Engineering, College of Design and Engineering, National University of Singapore, Singapore 117583

^5^ Department of Medical IT Convergence Engineering, Kumoh National Institute of Technology, Gumi, 39253, Republic of Korea

^6^ Institute for Health Innovation and Technology, National University of Singapore, Singapore 119276

The N.1 Institute for Health, National University of Singapore, Singapore 117456

^7^ Department of Surgery, Yong Loo Lin School of Medicine, National University of Singapore, Singapore 119228

^8^ Department of Biomedical Engineering, College of Engineering, City University of Hong Kong, Kowloon, Hong Kong SAR, China

* Authors to whom any correspondence should be addressed

Juwita Norasmara binte Rahmat

**E-mail:** [biejnr@nus.edu.sg](mailto:biejnr@nus.edu.sg)

Yong Zhang

**E-mail:** [yozhang@cityu.edu.hk](mailto:yozhang@cityu.edu.hk)

**Additional Methods**

**Characterization 405 LEDs induced RB release from RB-M**

To monitor the release of RB from the RB-M micelles, 0.2 mL RB-M (0.4 mg ml^-1^) was placed in one well of a 96-well plate followed by irradiation from the 405 nm LED (2 mW cm^-2^) for 60 min. The RB-M solution was then diluted to 2 mL with phosphate-buffered saline (PBS) solution and placed in a 3500 Da dialysis tub. For comparison, a similar RB-M solution without light irradiation was similarly diluted to 2 mL with PBS and placed in a 3500 Da dialysis tube. The dialysis bags were then immersed in 20 mL PBS solution with 0.1% Tween-80. The entire setup was gently shaken at 150 rpm at 37 ℃, and 4 mL of the release medium was taken out at predetermined time points and freeze-dried, and the same volume of PBS with 0.1% Tween-80 was added back to maintain a constant volume. The RB concentration in the release medium was measured by testing fluorescence of RB using a microplate reader (Infinite M200 PRO, Tecan Ltd., Switzerland) (Excitation (Ex) = 480 nm; Emission (Em) = 530 nm) with reference to a standard calibration curve of free RB dissolved in PBS.

**Fabrication of Wireless LED**

The wireless light delivery system requires sequential light control to break down the drug delivery carrier by irradiating it with light at 405 nm and activate the photosensitizer by irradiating it with light at 580 nm. The proposed dual-light delivery system, which is made up of independent wireless powering links with different resonant frequencies for each light source, is easily controllable by applying an external magnetic field with specific resonant frequencies. The wireless light delivery device is constructed by stacking two independent wireless LED modules, each containing a printed spiral coil, a half-wave voltage doubler, a rectifier for alternating current to direct current conversion, and two light-emitting diodes (LEDs). The half-wave voltage doublers are composed of capacitors (100-pF capacitor (06031A101JAT2A, AVX Corporation, USA), 220-pF capacitor (C0603C221K1RACTU, KEMET, USA), 27-pF capacitor (GCM0335C1H270JA16D, Murata Electronics, Japan), 39-pF capacitor (GRM0335C1E390FA01D, Murata Electronics, Japan) and Schottky diodes (BAT2402LSE6327XTSA1CT-ND, Infineon Technologies, Germany). All the electronics were mounted using a micro-soldering station (NAE-2A, JBC, USA) under a microscope (SZ61, Olympus, Japan) with lead-free soldering materials (SMD291SNL10, ChipQuik, USA). The electronics were coated by applying epoxy to prevent mechanical and moisture-induced damage. To prevent electrical shorts, an insulator layer has been inserted between a 580 nm (SML-P12Y2TT86R, Rohm Semiconductor, Japan) wireless LED module that is resonated at 50 MHz and a 405 nm (SM0603UV-400, Bivar Inc, USA) wireless LED module that is activated at 25 MHz. After degassing in a vacuum chamber for 30 minutes, the wireless LED device was encapsulated by PDMS with a 3D printed mold and baked in a forced convection oven (Esco Isotherm, SG) at 70 °C for 4 h. The encapsulated device was coated one more time with rapid curing, and a biocompatible silicone (WPI, Kwik-Sil, UK) to remove the remaining cracks on the surface. The dual-light delivery device, constructed of two wireless LED modules stacked together, was safely implanted in the abdominal region of the rat with less than 4 mm of thickness and 10 mm of size.

**Detection of ROS generated from RB-M under LED irradiation**

SOSG was used to detect the ROS generated under LED irradiation. Free RB or RB-M was diluted with PBS containing 4 µM of SOSG to an RB concentration of 5 µM. Then, the solutions were irradiated with the 405 nm LED, 580 nm LED, or 405 nm and 580 nm LEDs together for different durations. The fluorescence of the solution was then analyzed using an Infinite M200 PRO microplate reader ((Ex) = 480 nm; (Em) = 530 nm).

**Cell Culture and Viability Measurements**

McA-RH7777 (CRL-1601), N1S1 (CRL-1604), HepG2 (HB-8065), and HeLa (CCL-2) cell lines were obtained from American Type Culture Collection (ATCC, USA). All cell lines were kept within 20-25 passages after culture expansion from ATCC source. All media contained 10% FBS and 1% antibiotics. All cells were cultured in DMEM complete medium at 37℃ in a 10% CO_2_ incubator. To investigate the in vitro toxicity of RB-M with PDT treatment to cells, 1x10^4^ HeLa cells were seeded in 96-well plates. After 24 h, the original culture medium was replaced with fresh ones containing 0.02, 0.05, 0.1, 0.2, 0.5, 1, 2 mg mL^-1^ RB-M and further incubation for 12 h. There are two PDT lighting protocol programs: sequential PDT (580-405) means irradiation of 580 nm LED followed by the irradiation of 405 nm LED; sequential PDT (405-580) means irradiation of 405 nm LED followed by the irradiation of 580 nm LED. LEDs with two different PDT lighting protocols were irradiated at 12, 15, and 18 dBm for 20, 40, and 60 min. Then, the culture medium was replaced, and cells were left to incubate for another 24 h. Finally, 90 µL medium with 10 µL CCK8 kit (Sigma-Aldrich) was added to each well. The absorbance of each well was measured at 450 nm using a universal microplate reader (FLX800, USA).

**3D Spheroid Formation and RB-M Uptake**

McA-RH7777 and HepG2 cells were collected and counted with trypan blue, then diluted the cell suspension to obtain 5 ×10^3^ cells mL^-1^. Before incubation, 200 µL well was dispensed into the ultra-low attachment 96-well round bottom plates. The spheroids were acquired by light microscopic images every 2 days. After acquiring images, 100 µL of the old medium in each well was removed and replaced with 100 µL of fresh medium.

The HepG2 spheroids with similar size were selected and incubated with the sample (0.2 mg mL^-1^ RB-M) at varying time points (2 h, 12 h) in 37° environment. After this time, the media was removed, and spheroids were washed with cell medium. The RB-M content was analyzed using confocal scanning microscopy (Leica). Z-stack images with 1 µm interval were obtained by scanning the HepG2 spheroid layer-by-layer. The scanning began from the bottom of a spheroid. The fluorescent signal at each layer was calculated by Image J (NIH, USA).

**3D Spheroid ROS Detection and Live/Dead assay**

After 12 h incubation with 0.2 mg mL^-1^ RB-M, the HepG2 and McA-RH7777 spheroids were prepared and washed with fresh media in ultra-low attachment 96-well plates. Irradiation with the different PDT protocols followed, such as concurrent PDT (580+405), which meant irradiation of 580 nm LED and 405 nm LED together for 30 mins, sequential PDT (580-405) which meant irradiation of 580 nm LED followed by irradiation with 405 nm LED with 30 mins, and sequential PDT (580-405) which meant first irradiation of 580 nm LED with 30 mins following the irradiation of 405 nm LED with 30 mins. For sequential PDT, there is a 30 mins gap between the irradiations of the two light wavelengths. RB-M only group are treated with RB-M but without LED irradiation. The control group only contained the spheroids without RB-M and LED irradiation.

The green reactive oxygen species detection kit (DCFH-DA, ThermoFisher) was prepared and added to the wells before PDT treatment. After treatment, the HepG2 spheroids were labeled with Hoechst 33342 and incubated for 20 mins. Then, the spheroids were washed with PBS. The fluorescent signal of each spheroid was recorded at excitation 488nm for DCFH and excitation 350 nm for Hoechst 33342 using fluorescent microscopy (Olympus). As for the spheroid viability, the calcein AM and ethidium homodimer-1 from live/dead kit (ThermoFisher) were obtained and added into each well to stain the HepG2 and McA-RH7777 spheroids after finishing the PDT treatment as mentioned above. Then, the spheroids were rinsed with PBS and prepared for fluorescent imaging. The fluorescent signal of each spheroid was recorded at excitation 494 nm for calcein AM and excitation 528 nm for ethidium homodimer-1. Quantitative analysis of fluorescent signal was calculated by Image J (NIH, USA).

**3D Spheroid Proliferation, Invasion, and Migration assay**

*Anchorage-independent proliferation*: McA-RH7777 cell suspension was collected and implanted with 200µL 5 ×10^3^ 3 cell/mLinto each well of ultra-low attachment 96-well plates. After 2 h incubation with RB-M, the PDT commenced according to the respective group light programs. Then, the supernatant was removed by centrifugation. Matrigel (0.2 mL, 0.2 mg mL^-1^ ) was mixed with the complete medium and added to each well. Microscopic images of each well were taken daily ^[1]^.

*Spheroid invasion assay*: A collagen matrix with type I collagen at 1 mg mL^-1^ final concentration in 1× PBS was prepared and transferred into the bottom of 24-well plates. The plates were incubated in a 37°C incubator until the wells dried. McA-RH7777 spheroids were washed with PBS, carefully pipetted into the collagen matrix, and transferred in the center of the well of the 24-well plates. Then, the collagen gel with spheroids was incubated for 30 min at 37℃, and complete medium was added on top of the gel. RB-M PDT treatment was then performed following the respective light programs. Microscopic images of each spheroid were taken every 2 days after collagen inclusion.

*3D spheroid migration*: Matrigel (0.2 mg mL^-1^ ) in complete medium was coated onto a 24-well plate and incubated for 30 min at 37℃. The McA-RH7777 spheroids were washed with PBS and carefully transferred into a complete medium from the round bottom well plate to the 24-well plate. The plate was incubated at 37℃ for 30 min for the spheroids to adhere. RB-M PDT treatment was then performed following the respective light programs. Microscopic images of each spheroid were taken every 2 days.

The anchorage-independent proliferation area, invasion distance, and migration distance were analyzed manually. The anchorage-independent proliferation area was measured by the total area of the spheroid under the same field of view. The metastasis distance of each spheroid was measured by the difference between the farthest distance from spheroids and the semidiameter of the spheroid using the in-built microscope software in brightfield.

**Generation of N1S1-luc for in vivo tumor implantation**

pLVX-Luc-puro was cloned in house, under a CMV promoter. MD2G and psPax2 packaging plasmids were kind gifts from Didier Trono (EPFL). Viral particles carrying firefly luciferase (Luc) were generated according to standard protocol. Briefly, Lenti-X 293T cells were transfected with vectors carrying gene of interest, psPax2 and MD2G with Lipofectamine 2000 reagents, according to the manufacturer’s protocol. The viral supernatants were sterilized with 0.45um filter and used for transduction of N1S1 cells. The transduced N1S1 cells were selected with puromycin (1ug/mL) and maintained in DMEM complete medium at 37℃ in a 10% CO_2_ incubator.


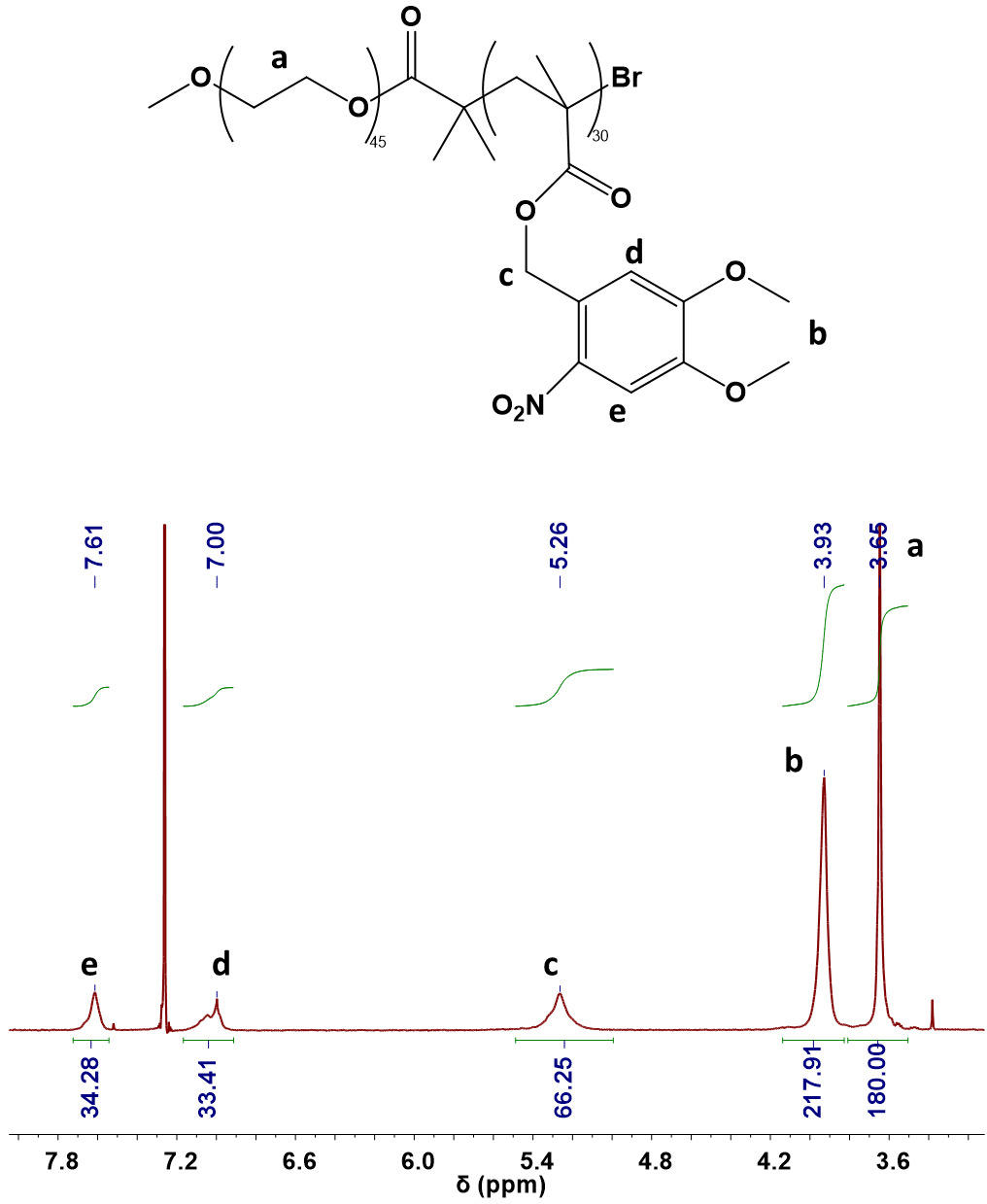


**Fig. S1.** Chemical structure and ^1^H NMR spectrum of PEG45-b-PNMA30 block polymer

**Fig. S2**. DLS size distribution of RB-M dispersed in water (by intensity).


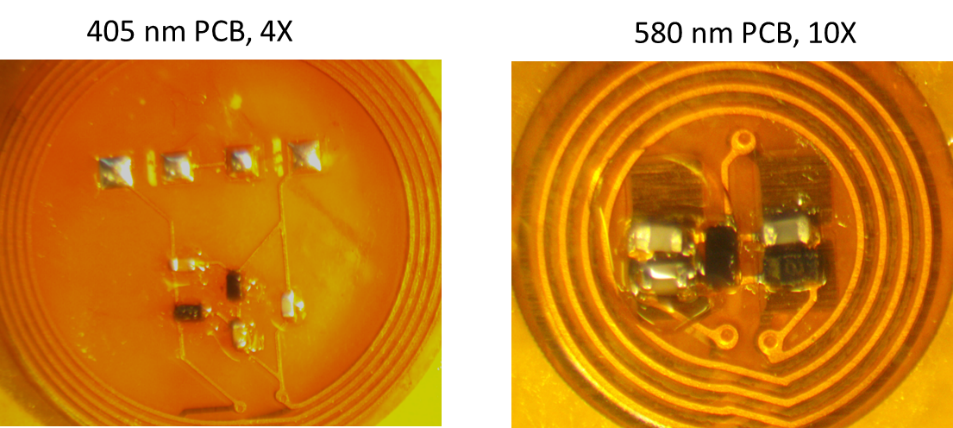


**Fig. S3**. Construction and layout of LED PCB, including capacitors, Schottky diode, and four LEDs with the same specification.


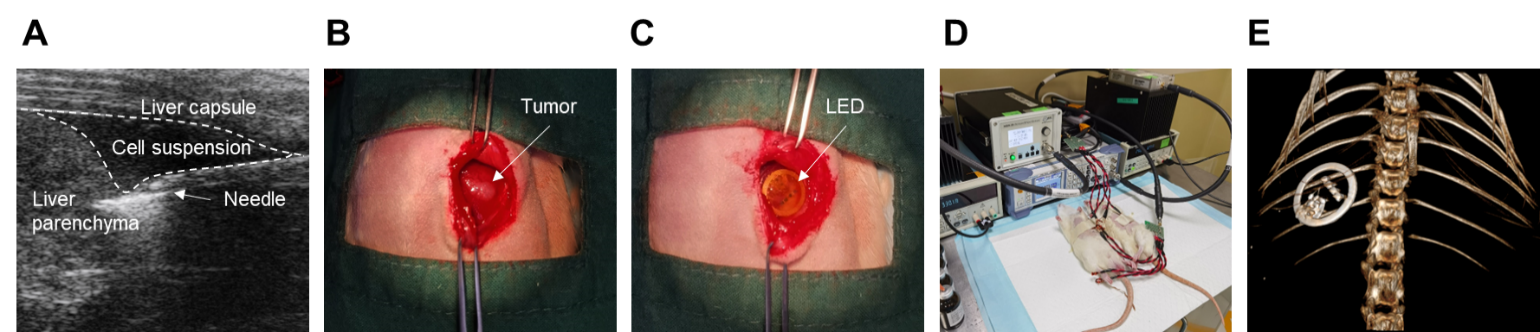


**Fig. S4**. The procedures of RB-M PDT treatment and RB-M distribution in vivo. The representative images of animal experiments treatment from left to right: (A) ultrasound image of tumor cells implantation, (B) surgical incision to access tumor tissue, (C) surgical implantation of wireless LED device, (D) irradiation for PDT, and (E) LED positioning check with CT scan.


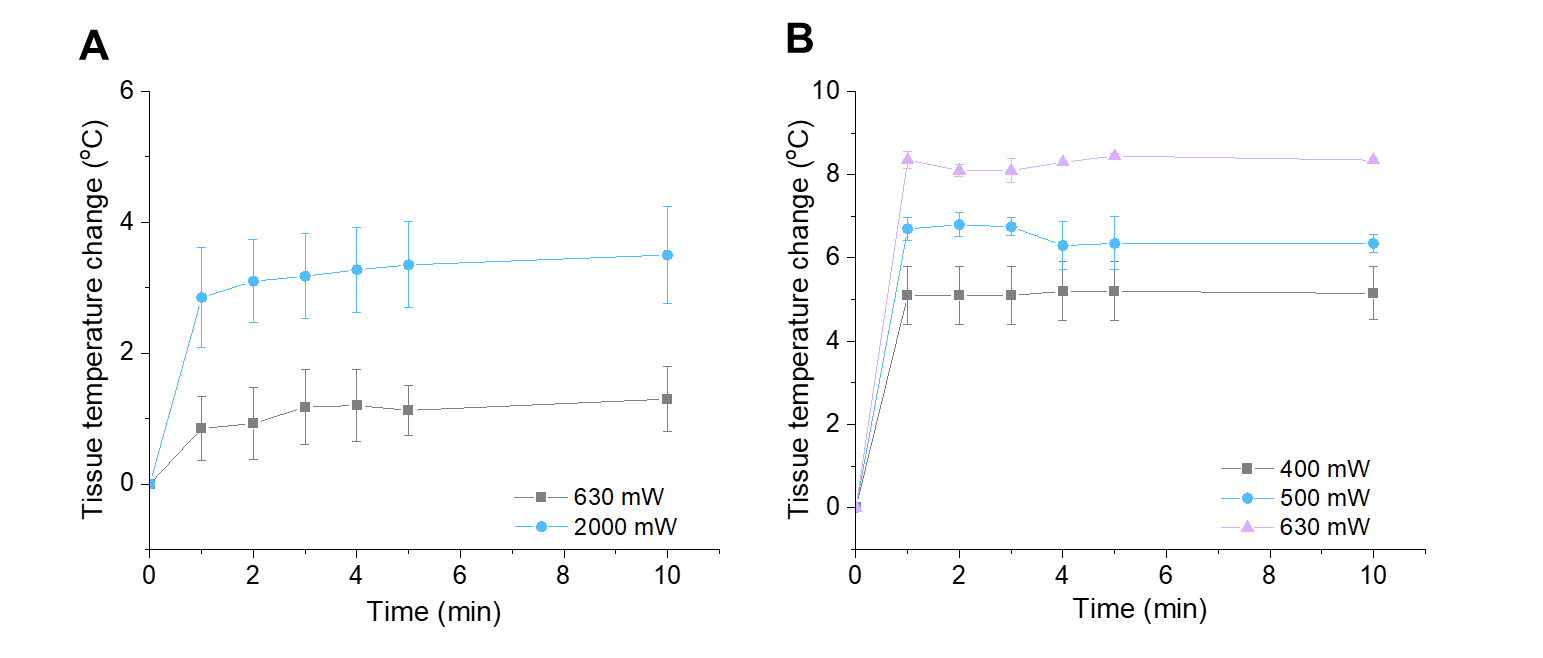


**Fig. S5**. Tissue temperature change after wireless powering of (A) 405 nm LEDs and (B) 580 nm LEDs. Data expressed as mean ± SD. The experiment was done twice (n=4)


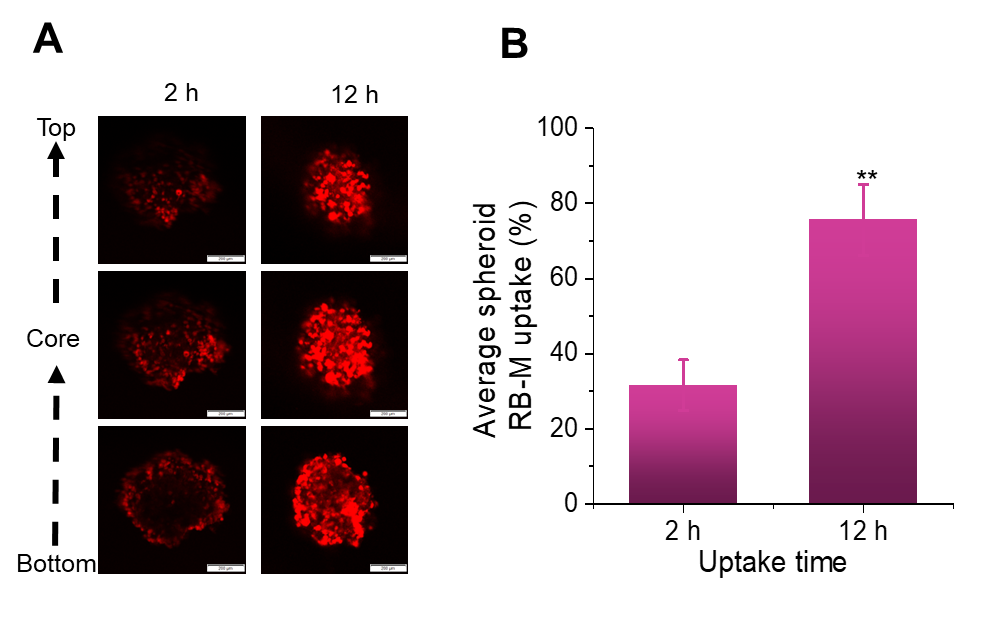


**Fig. S6**. The uptake of RB-M nanoparticles in HepG2 3D spheroids. (A) Detection of cellular ROS in HepG2 spheroids after RB-M PDT with varying light programs. (B) The uptake of RB-M in HepG2 spheroids. The spheroids were treated with 0.2 mg mL^-1^ RB-M, and the penetration of RB-M in the spheroid core was detected with confocal microscopy after 2 and 12 h. A z-stack slicing was performed to detect the RB-M penetration at varying spheroid depth. Scale bar, 200 µm. Data represented as mean ± SD. * p< 0.05, ** p< 0.01.


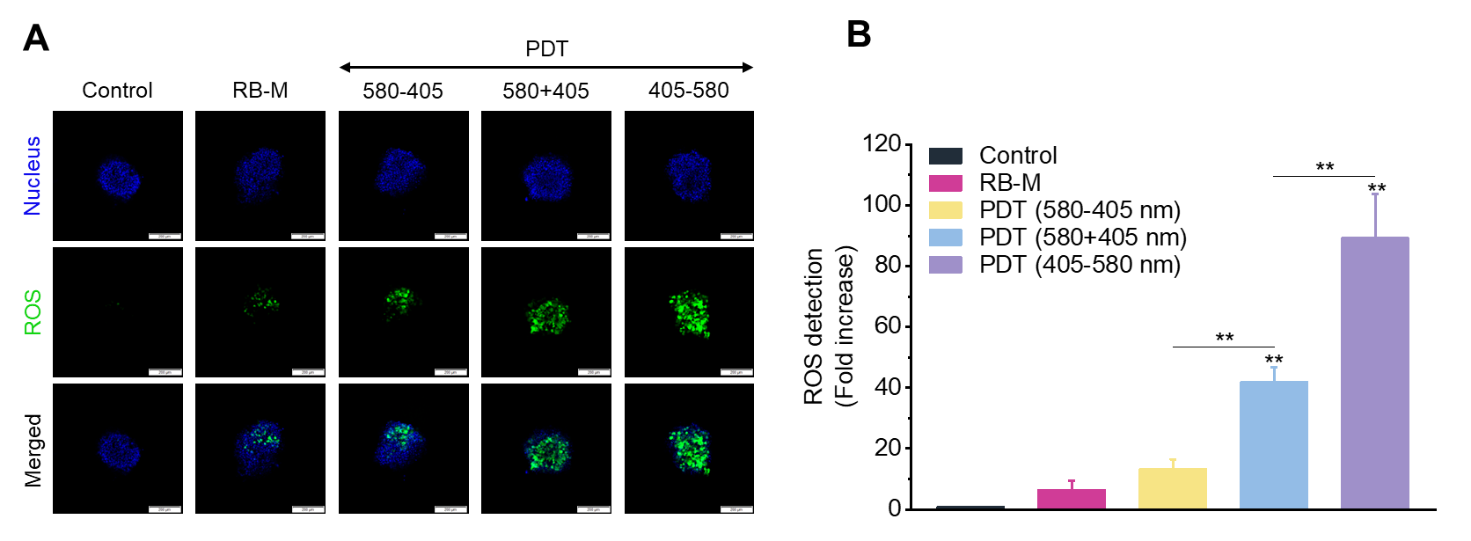


**Fig. S7**. ROS generated by various light programs after PDT with RB-M. (A) Detection of ROS generated via confocal microscopy after PDT with RB-M. (B) Quantification of ROS fluorescence levels. Scale bar, 200 µm. Data represented as mean ± SD. * p< 0.05, ** p< 0.01.


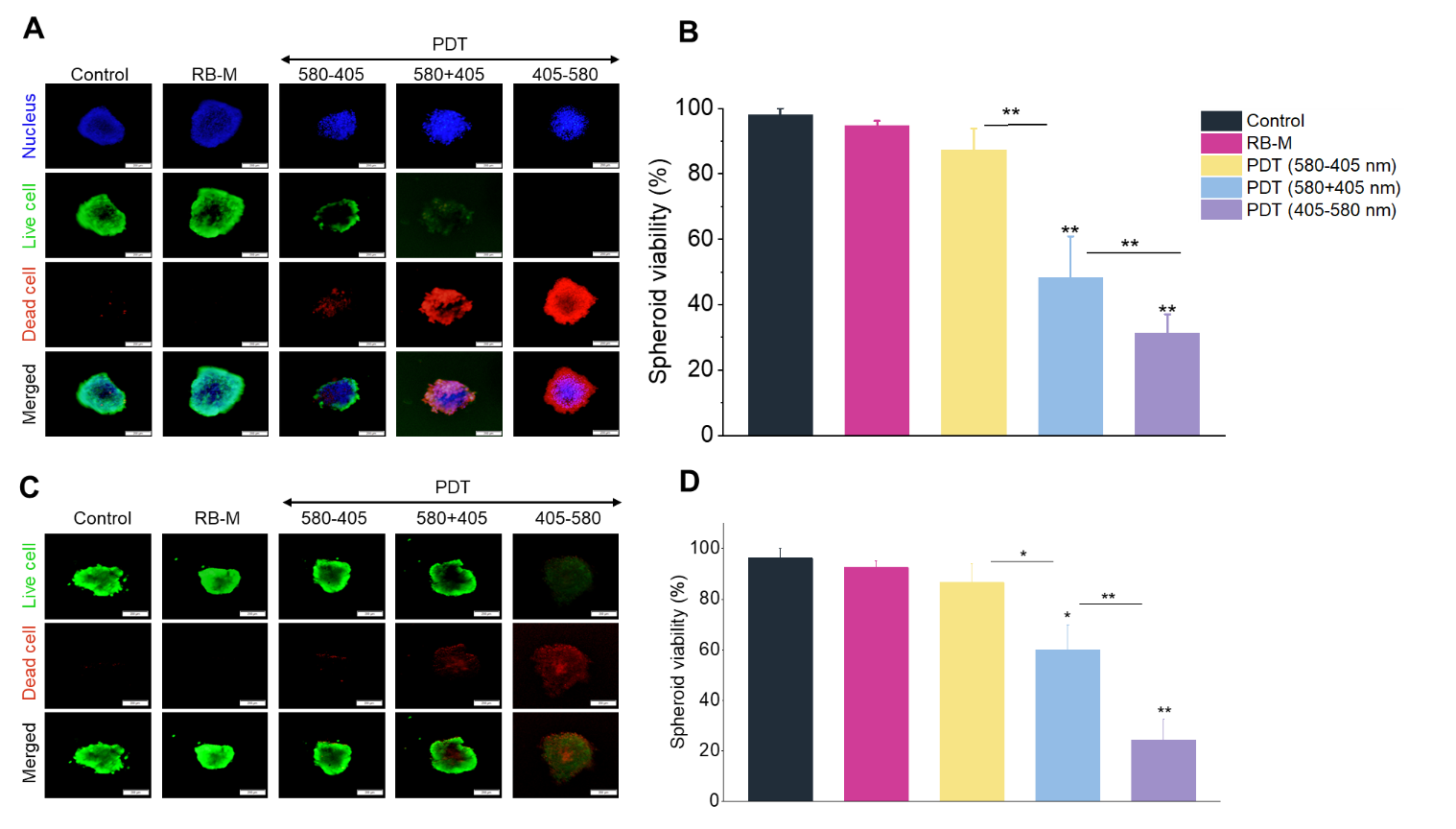


**Fig. S8**. Cytotoxic effects of various light programs after PDT with RB-M. (A) Live/dead staining assay with HepG2 spheroids. (B) Quantification of HepG2 spheroid viability after PDT with various light programs. (C) Live/dead staining assay with McA-RH7777 spheroids after RB-M PDT using various light programs. Scale bar, 200um. (D) McA-RH7777 spheroid viability after PDT with various light programs. Scale bar, 200 µm. Data represented as mean ± SD. * p< 0.05, ** p< 0.01.


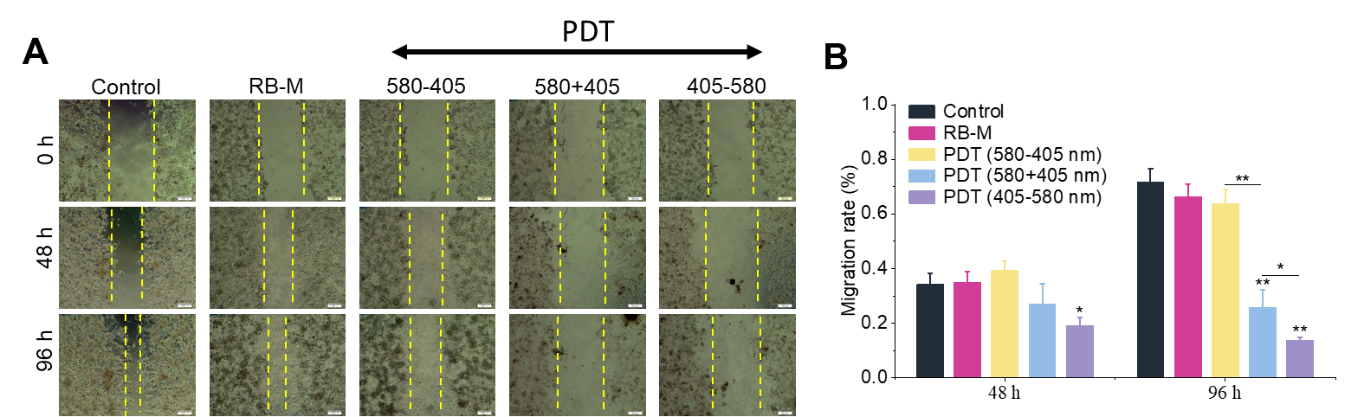


**Fig. S9. (**A) Bright-field microscopy images of Mca-RH7777 monolayer cells at 0, 48 and 96 hrs after PDT with various light programs. The dashed yellow line at Day 0 indicates the gap generated with the edge of a yellow pipette tip. At 48 hr and 96 hr, the dashed yellow line indicates the closure of the gap after PDT. (B) The average migration rate of the cells in the gap periphery after PDT. Scale bar, 200 µm. Data represented as mean ± SD. * p< 0.05, ** p< 0.01.


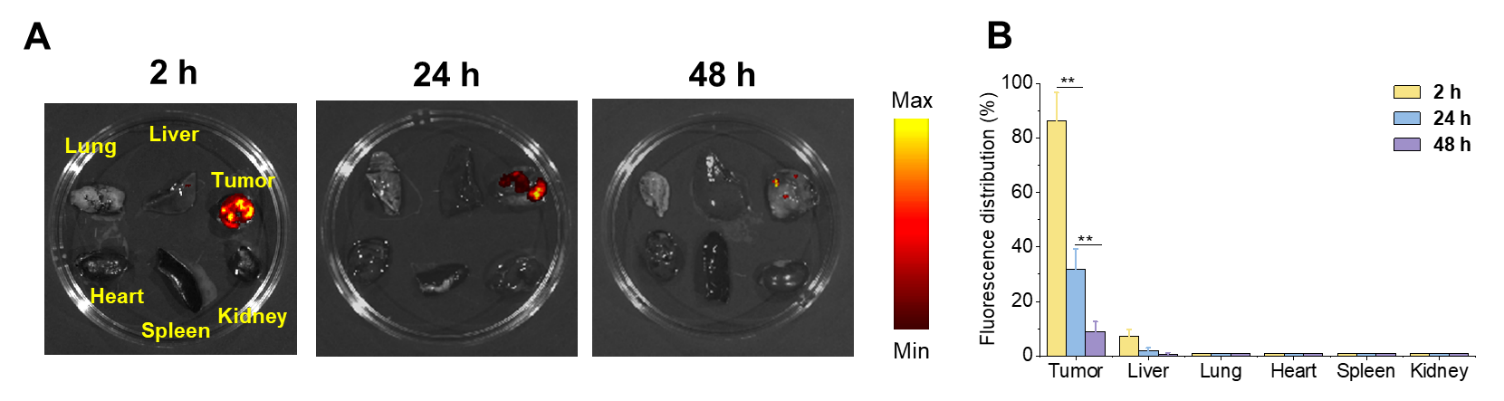


**Fig. S10.** (A) RB-M biodistribution after 2, 24, and 48 h. (B) Quantitative analysis of fluorescence signal intensity at different organs. * p< 0.05, **p<0.01.

**References**

1 S. Mori, J. T. Chang, E. R. Andrechek, N. Matsumura, T. Baba, G. Yao, J. W. Kim, M. Gatza, S. Murphy, and J. R. Nevins, *Oncogene*, **2009**, 28, 2796
